# Secondary Hemochromatosis Caused by Iron Overdose During Pregnancy and the Postpartum Period: A Case Report

**DOI:** 10.7759/cureus.64355

**Published:** 2024-07-11

**Authors:** Miho Shishii, Shunsuke Hyuga, Masashi Miyamoto, Noriko Terada, Waso Fujinaka

**Affiliations:** 1 Department of Anesthesiology and Intensive Care, Hiroshima City Hiroshima Citizens Hospital, Hiroshima, JPN; 2 Department of Obstetric Anesthesia, Center for Perinatal Care, Child Health and Development, Kitasato University Hospital, Hiroshima, JPN

**Keywords:** differential diagnosis, perinatal hepatic dysfunction, pregnancy, iron deficiency anemia, secondary iron overload

## Abstract

Iron deficiency anemia is the most common cause of anemia in pregnancy. Therefore, iron administration is recommended for treatment. Iron deficiency anemia during pregnancy does not always result in microcytic anemia. Thus, iron may continue to be administered as diagnostic therapy, even in patients with normocytic anemia. In the present case, although the patient had normocytic anemia, repeated intravenous iron administration resulted in liver dysfunction due to secondary iron overload, which required intensive care. In pregnant women with perinatal hepatic dysfunction, iron overload secondary to iron therapy administered to correct anemia during pregnancy should be considered in the differential diagnosis.

## Introduction

Correction of iron deficiency anemia (IDA) during pregnancy is important because it is associated with low birth weight, preterm delivery, and increased perinatal mortality [1]. IDA is treated with iron supplementation, which is recommended worldwide [1-3]. However, physiological changes associated with pregnancy may make diagnosis difficult, and iron may be administered as a diagnostic treatment [3]. Conversely, secondary iron overload involves excessive iron accumulation, causing liver disease, heart failure, diabetes mellitus, and other organ damage due to excessive iron absorption and intake after massive blood transfusions or in patients with erythropoietic disorders. We report a rare case of hepatic dysfunction, electrolyte abnormalities, and ascites due to iron overload secondary to intravenous iron administration for the treatment of anemia during pregnancy, which required intensive care management.

An abstract of this paper was presented at the 49th Annual Meeting of the Japanese Society of Intensive Care Medicine (2022, web).

## Case presentation

A 34-year-old primigravida (with twins, height: 159 cm, weight 48.1 kg (47 kg before pregnancy)) had unremarkable medical and family histories. At 14 weeks gestation, she had severe hyperemesis gravidarum and lost 16% of her nonpregnant weight (body mass index, 15.6 kg/m^2^). She was admitted to the hospital at 22 weeks and 5 days of gestation due to impending preterm labor. Table 1 shows the perinatal blood findings. At 25 weeks and 3 days of pregnancy, she was started on oral sodium ferrous citrate 100 mg/day for anemia; however, after two days, she could not continue because of hyperemesis gravidarum, and iron oxide 80 mg/day was administered intravenously until 27 weeks and 4 days of pregnancy. She gained 4 kg of body weight per week starting from 32 weeks and 3 days of gestation, and generalized edema appeared. Her blood pressure was 112/75 mmHg; as there was no proteinuria, preeclampsia was ruled out. Blood tests did not suggest HELLP (hemolysis, elevated liver enzymes,and low platelet count) syndrome. Thoracoabdominal ultrasonography revealed pleural effusion and ascites. Transthoracic echocardiography demonstrated normal cardiac function, ruling out heart failure. At 33 weeks and 2 days of gestation, 24-hour creatinine clearance was 35 mL/min, indicating renal dysfunction without hypertension or proteinuria. Therefore, an urgent cesarean section was performed following the decision of the obstetrician based on the organ load associated with the twin pregnancies due to worsening hepatic and renal function evident from a blood test (aspartate aminotransferase (AST) 78 IU/L, alanine aminotransferase (ALT) 54 IU/L, creatinine 0.7 mg/dL) at 34 weeks and 2 days of gestation weeks of gestation.

**Table 1:** Changes in perinatal blood test findings POD: postoperative day; Hb: hemoglobin; MCV: mean corpuscular volume; Plt: platelet; Fe: serum iron; Fr: ferritin; TIBC: total iron-binding capacity; AST: aspartate aminotransferase; ALT: alanine aminotransferase; LDH: lactic acid dehydrogenase; CRP: C-reactive protein; BUN: blood urea nitrogen; CRE: creatinine; 24hCcr: 24-hour creatinine clearance; Alb: albumin; PreAlb: prealbumin; K: potassium; Mg: magnesium; IP: inorganic phosphorus; Zn: zinc

Inspection item	Inspection period									
	25w3d	32w2d	33w2d	34w2d	POD4	POD6	POD8	POD10	POD13	POD27
Hb (g/dL)	9.2	10.4	11	11.6	9	6.3	6.4	6.1	5.9	10.4
MCV (fL)	97.8	94.6	92	91.6	92.3	92.8	95.9	97.8	101.1	104.2
Plt (10^4^/μL)	17.4	8.3	9.9	8.4	10.4	4.4	9.3	12.4	22.3	43.5
Fe (μg/dL)	64	-	-	-	-	171	82	43	58	55
Fr (ng/mL)	17.6	-	-	-	-	10494	3760	1702	833	484
TIBC (μg/dL)	-	-	-	-	-	166	-	-	-	-
AST (IU/L)	22	43	57	78	59	646	166	56	30	29
ALT (IU/L)	12	25	34	54	17	352	216	118	59	25
LDH (IU/L)	193	378	447	580	441	979	463	406	-	304
CRP (mg/dL）	0.012	0.024	0.033	0.074	0.236	1.911	0.96	0.495	0.318	0.032
BUN (mg/dL)	9	17	28	37	31	17	15	10	11	11
CRE (mg/dL)	0.42	0.52	0.58	0.7	0.54	0.36	0.27	0.29	0.27	0.33
24hCcr (ml/min)	-	-	35	-	-	-	-	-	-	-
Alb (g/dL)	-	-	-	-	-	2.1	-	-	1.9	2.7
PreAlb (mg/dL)	-	-	-	-	-	10	-	-	10	23
K (mEq/L)	3.6	4.5	4.8	4.6	3.5	2.8	4.5	4.2	-	3.7
Mg (mg/dL)	-	-	-	-	-	1.9	2.2	-	-	2
IP (mg/dL)	-	3.5	3.6	4.8	-	0.8	3.3	3.2	-	3.2
Zn (μg/dL)	-	-	-	-	-	26	-	-	50	59

**Figure 1:**
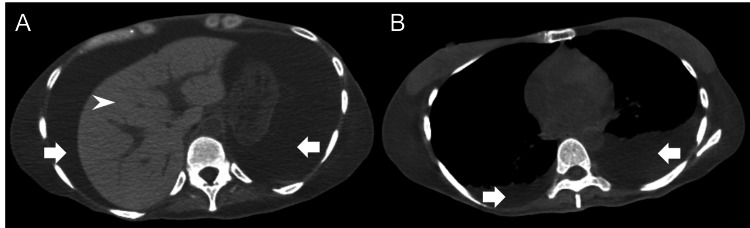
Simple computed tomography (CT) (A) High-density area (73 Hounsfield Units, HU) of the liver parenchyma (white arrowhead), with no gross liver mass visible. (A, B) Thoracoabdominal fluid (white arrow). Chronic inflammatory disease causing hyperferritinemia was not identified.

The operation was performed under spinal anesthesia, with an intraoperative blood loss of 1,390 g. On postoperative day (POD) 4, the patient became anemic, and intravenous administration of 80 mg/day of iron oxide-containing sugar was resumed. On POD 6, liver enzymes were elevated, and electrolyte abnormalities were observed (Table 1). Physical examination revealed abdominal distention, with ascites noted on abdominal ultrasonography, and she was admitted to the intensive care unit (ICU). Blood tests showed normocytic anemia, with iron 171 μg/dL (reference range: extracted from the facility standards 40-188 μg/dL), ferritin 10,494 ng/mL (5.0-152.0 ng/mL), and total iron binding capacity of 166 μg/dL (246-410 μg/dL). There was concern that chronic inflammation might be caused by an acute phase protein ferritin. Computed tomography (CT) showed hyperabsorption of liver parenchyma, and since a source of inflammation could not be identified (Figure 1), iron overload secondary to excessive iron administration was diagnosed. Blood tests showed Child-Pugh classification grade B (9 points) liver dysfunction and decreased protein synthesis; CT showed left pleural effusion and ascites effusion, but she did not have dyspnea, and her breathing was normal. We treated her with furosemide and spironolactone without oxygen therapy. After ICU admission, electrolyte correction was started with intravenous potassium chloride, magnesium sulfate, sodium phosphate, zinc acetate hydrate, and oral polaprezinc. Intravenous nutrition was started at 10 kcal/kg/day of carbohydrate, and caloric intake and protein load were increased stepwise. As a treatment for iron overload, discontinuation of iron administration, including blood transfusions and diet from the day of ICU admission, resulted in ferritin 3,760 ng/mL on POD 8. Ferritin did not increase, and she left the ICU on POD 16. On POD 27, protein synthesis improved, and ascites decreased with time. After leaving the ICU, she was discharged on POD 42 after laboratory data showed improvement.

## Discussion

Anemia in pregnancy is defined as a hemoglobin level of <11.0 g/dL (hematocrit <33%). A British survey reported that 46% of women were anemic at some point during pregnancy [4,5]. The most common cause is IDA, which is associated with low birth weight, preterm delivery, and increased perinatal mortality if untreated [1,2]. Therefore, Japanese obstetric practice guidelines [2] and European and American perinatal obstetric and hematology guidelines [1,3] recommend aggressive correction of anemia during pregnancy with iron supplements. However, IDA during pregnancy may not result in microcytic hypochromic anemia, as maternal hemoglobin volume does not normally increase while systemic plasma volume increases and the mean corpuscular volume (MCV) is often slightly low or normal [6]. Even when MCV is normocytic, as in this case, IDA cannot be ruled out, and iron may be administered as a diagnostic treatment [1,3,7]. Measurement of serum ferritin level is useful in diagnosing IDA. In pregnancy, ferritin level (in some studies, <30 ng/mL) is the gold standard for IDA diagnosis, as it is the most sensitive and specific indicator [1,3]. However, not all facilities measure ferritin levels before starting iron administration due to the characteristics and prevalence of iron metabolism during pregnancy, and some guidelines recommend iron administration for a diagnostic trial [1,3].

Thus, aggressive administration of iron was performed, and the patient being unable to initially receive iron orally due to hyperemesis gravidarum and intravenous administration may have contributed to the severe iron overload because all the iron entered the bloodstream. Furthermore, the progression of anemia on POD 4 was not attributed to acute hemorrhage based on examination findings. Therefore, the patient should have been assessed prior to iron administration, but treatment was resumed based on the clinical diagnosis of IDA without close examination. Given the association between postpartum anemia and postpartum depression, the preoperative iron administration was repeated [8]. Consequently, an unintended medical iron overload ensued due to continuous and excessive iron administration, which is regrettable. It is important to check ferritin levels using periodic blood tests before and after administration rather than just administering the drug [3]. There are two types of iron overload: primary (with a genetic predisposition) and secondary (associated with anemia, resulting in ineffective hematopoiesis or iron overdose). Over 90% of the cases are due to long-term massive erythrocyte transfusion [9], causing organ damage, including liver injury, diabetes mellitus, and heart failure [10]. Although a reliable diagnosis is made by measuring liver iron concentration, in this case, platelet count was decreased (44,000/µL at POD 6 on admission to the ICU) and a liver biopsy was invasive and could not be performed. Serum ferritin level and total iron binding capacity are important indices in blood tests [11], and the serum ferritin level is particularly useful in predicting the disease severity and the occurrence of complications [9]. CT, magnetic resonance imaging, and other imaging modalities can also evaluate the liver iron stores. Liver brightness increases on CT images in patients with severe iron overload [12], and liver CT values correlate well with serum ferritin levels and body iron stores, with some reports indicating severe iron deposition above 72 HU [13]. Usually, diseases indicating liver dysfunction during pregnancy include pre-eclampsia, HELLP syndrome, and acute fatty liver of pregnancy. The findings of elevated liver enzymes, renal dysfunction, elevated lactic acid dehydrogenase, and decreased platelet count in our case are consistent with those diseases. However, pre-eclampsia and HELLP syndrome itself usually do not have characteristic liver imaging findings [14], and in acute fatty liver of pregnancy, there is usually no increased resorption of the liver parenchyma as in this case, although CT may show decreased attenuation of the liver [15]. In this case, in addition to a history of intravenous iron administration, blood tests showed elevated ferritin levels, decreased total iron binding capacity, and impaired iron utilization, and a simple CT scan showed hyperabsorption of the liver parenchyma, suggesting iron overload. Despite the limitation that a liver biopsy was not available as a definitive diagnosis, the diagnosis of iron overload secondary to excessive iron administration was made based on other characteristic findings.

Treatment of iron overload involves phlebotomy and administration of iron chelating agents, depending on the condition and disease severity [12]. The timing of treatment initiation should be determined considering organ damage and prognostic impact. However, guidelines from various countries consider a ferritin level of 1,000 ng/mL as an index for starting iron chelation therapy in patients with secondary iron overload [16,17]. In this case, the ferritin level was 10,494 ng/mL at ICU admission, a level at which treatment with iron-chelating agents should be considered at the earliest. However, the ferritin level at that time was measured under conditions that could have resulted in a falsely high ferritin level due to several factors, including the patient just receiving intravenous iron and having renal dysfunction [17,18]. Considering the side effects of the iron-chelating agent including gastrointestinal and renal dysfunction, we followed up with the patient under strict control with daily follow-up of ferritin levels. The patient recovered spontaneously after discontinuing all iron administration, including blood transfusions and diet. Thus, no iron-chelating agent was required.

## Conclusions

In summary, we encountered a case of secondary iron overload due to repeated intravenous iron administration to correct anemia during pregnancy and postpartum. Although IDA in pregnant women is frequent and may not meet the diagnostic criteria on blood tests due to physiological changes associated with pregnancy, treatment with iron may be administered without close examination because correction of anemia during pregnancy is recommended. Evaluation of ferritin levels and iron should be repeated after intravenous iron administration or upon resumption of therapy. In pregnant women with hepatic dysfunction, perinatal disease is often considered the cause. However, iron overload secondary to iron overdose should be ruled out if the patient was administered iron.

## References

[1] (2021). Anemia in pregnancy: ACOG Practice Bulletin, Number 233. Obstet Gynecol.

[2] Nishio E, Ishitani K, Arimoto T (2024). Guideline for gynecological practice in Japan: Japan Society of Obstetrics and Gynecology and Japan Association of Obstetricians and Gynecologists 2023 edition. J Obstet Gynaecol Res.

[3] Pavord S, Daru J, Prasannan N, Robinson S, Stanworth S, Girling J (2020). UK guidelines on the management of iron deficiency in pregnancy. Br J Haematol.

[4] Pasricha SR, Colman K, Centeno-Tablante E, Garcia-Casal MN, Peña-Rosas JP (2018). Revisiting WHO haemoglobin thresholds to define anaemia in clinical medicine and public health. Lancet Haematol.

[5] Benson CS, Shah A, Frise MC, Frise CJ (2021). Iron deficiency anaemia in pregnancy: a contemporary review. Obstet Med.

[6] Cunningham FG, Leveno KJ, Dashe JS, Hoffman BL, Spong CY, Casey BM (2022). Williams Obstetrics. 26th ed. Dashe JS, Hoffman BL, Spong CY, Casey BM: Williams Obstetrics. 26th ed.: McGraw-Hill Education 1304.

[7] Haram K, Nilsen ST, Ulvik RJ (2001). Iron supplementation in pregnancy - evidence and controversies. Acta Obstet Gynecol Scand.

[8] Maeda Y, Ogawa K, Morisaki N, Tachibana Y, Horikawa R, Sago H (2020). Association between perinatal anemia and postpartum depression: a prospective cohort study of Japanese women. Int J Gynaecol Obstet.

[9] Ikuta K, Hatayama M, Addo L (2017). Iron overload patients with unknown etiology from national survey in Japan. Int J Hematol.

[10] Fleming RE, Ponka P (2012). Iron overload in human disease. N Engl J Med.

[11] de Montalembert M, Ribeil JA, Brousse V (2017). Cardiac iron overload in chronically transfused patients with thalassemia, sickle cell anemia, or myelodysplastic syndrome. PLoS One.

[12] Suzuki T (2017). Grant-in-aid for scientific research on intractable diseases. Research on idiopathic hematopoietic disorders (FY2008). Medical guide for posttransfusion iron overload disease. Nihon Naika Gakkai Zasshi.

[13] Howard JM, Ghent CN, Carey LS, Flanagan PR, Valberg LS (1983). Diagnostic efficacy of hepatic computed tomography in the detection of body iron overload. Gastroenterology.

[14] Westbrook RH, Dusheiko G, Williamson C (2016). Pregnancy and liver disease. J Hepatol.

[15] Castro MA, Ouzounian JG, Colletti PM, Shaw KJ, Stein SM, Goodwin TM (1996). Radiologic studies in acute fatty liver of pregnancy. A review of the literature and 19 new cases. J Reprod Med.

[16] Bennett JM (2008). Consensus statement on iron overload in myelodysplastic syndromes. Am J Hematol.

[17] Blunden RW, Lloyd JV, Rudzki Z, Kimber RJ (1981). Changes in serum ferritin levels after intravenous iron. Ann Clin Biochem.

[18] Dignass A, Farrag K, Stein J (2018). Limitations of serum ferritin in diagnosing iron deficiency in inflammatory conditions. Int J Chronic Dis.

